# Intradialytic Parenteral Nutrition in Pediatrics

**DOI:** 10.3389/fped.2018.00267

**Published:** 2018-10-04

**Authors:** Marisa Danielle Juarez

**Affiliations:** Texas Children's Hospital, Houston, TX, United States

**Keywords:** intradialytic parenteral nutrition, end stage renal disease, pediatrics, hemodialysis, malnutrition

## Abstract

Children with end-stage renal disease (ESRD) on hemodialysis are at increased risk for malnutrition. Aggressive nutrition intervention such as intradialytic parenteral nutrition (IDPN) should be considered to prevent further co-morbidities and mortality associated with malnutrition when other interventions fail. IDPN is a non-invasive method of providing nutrition to malnourished hemodialysis (HD) patients via the HD access throughout the HD treatment. Although the evidence on the long-term benefits of IDPN is scant in pediatrics, there is evidence that it improves metabolic parameters and nutritional status. In this paper, therapy with IDPN including indications, goals of therapy, and elements to monitor will be described. In addition, a practice guideline for prescribing IDPN is provided.

## Introduction

### Malnutrition and growth failure in pediatric ESRD

Patients on hemodialysis (HD) are at increased risk for malnutrition (1–10). Poor intake due to anorexia of disease and/or oral aversion, poor tolerance of feedings, and protein degradation during HD with exposure to the dialyzer (11) are thought to lead to a catabolic state. In children, growth during the first 3 years of life is critical and highly influenced by nutritional intake (12–15). The Kidney Disease Outcomes Quality Initiative (KDOQI) and others recommend early nutrition supplementation in children if nutrition needs are not met and expected growth rates are not attained (15, 16). The majority of younger pediatric patients require enteral feeding to ensure adequate nutrition intake for age appropriate growth (12–17). According to the International Pediatric Peritoneal Dialysis Network (IPPN) 41% of young children on peritoneal dialysis require enteral feeds to meet nutritional needs (12). The IPPN studies and others have shown enteral feeding to improve growth parameters in chronic kidney disease (CKD) and ESRD compared to those fed orally (12, 14, 18). Enteral supplementation may be more successful in younger children by use of gastrostomy tube (GT). GT is preferred for long-term feeding over nasogastric tube due to the high prevalence of associated gastroesophageal reflux and vomiting from the NG tube. Furthermore, a GT allows for the development of normal oral motor skill development (12, 13, 17) and offers a route to provide medications without the stress of providing them orally (13). In older children however, the situation becomes more complex as body image, peer pressure, and taste acceptance influence compliance of enteral supplements and the multi-restricted diet omits many of the preferred foods of children. The adult literature recommends carefully choosing oral supplements based on patient smell and taste acceptance in order to improve compliance (19). Patients with ESRD however also suffer from anorexia. The etiology of which is unclear, but thought to be related to uremia, taste alterations, medications, and elevated cytokines (13).

The HD treatment is a catabolic event. Exposure to the dialyzer results in clearance of small and middle sized molecules such as proteins leading to a negative protein balance at the end of the HD treatment. Several adult studies show an association of weight loss and low serum albumin with mortality (19, 20). Similar results have been found in children. Wong et al. evaluated children with ESRD using data from the United States Renal Data System (USRDS) and found a 90% greater risk of death in patients with serum albumin <3.5 g/dL compared to those with albumin >3.5 g/dL (21). In another study, using data from the Pediatric Growth & Development Special Study (PGDSS) from the USRDS, Wong et al. found that anthropometric measures, specifically height, growth velocity, and extremes in body mass index (BMI), were significant predictors of death in both dialysis and transplant patients (22). For each decrease by 1 standard deviation score (SDS) in height and growth velocity, there was an increased risk of death by 14 and 12%, respectively. For BMI, there was a U-shaped association with increased risk of death, meaning both low and high BMI were associated with an increased risk of death (*p* = 0.001): 6% with ±1 SDS, 26% with ±2 SDS, and 67% with ±3 SDS (22). This is in contrast to adult studies which associate a higher BMI as protective from death (23, 24). Recognizing that in pediatrics the greater the severity of malnutrition, the greater the risk of death, supports the use of an intensified nutrition regimen such as IDPN to reverse malnutrition.

The definition of growth failure must be understood in order to appropriately identify malnutrition. According to Janjua and Mahan, growth failure is (25):

- <-1.88 SDS for age and gender for weight and/or height which is equivalent to <3rd percentile
- BMI <3rd percentile which suggests growth impairment and potential issues with weight and/or height-for-age in the future

Growth failure and CKD yield poor outcomes. Furth et al. found an astounding 2-fold increased risk of death for those with severe growth failure indicated by height-for-age <1% percentile or Z-score <-2.5 in the North American Pediatric Renal Trials and Collaborative Studies (NAPRTCS) data set (26). Approximately 35% of children with CKD have severe growth failure at the pre-dialysis stage (27). Many factors are associated with poor growth in children with CKD (Table 1). In comparison, nutrition barriers in ESRD are profound. Aggressive nutrition intervention such as IDPN should be considered to prevent further co-morbidities and mortality associated with malnutrition (3–7, 10, 16, 28, 29). Adult studies have shown improvement in anthropometrics and nutrition markers with use of IDPN; however, the lack of or incomplete coverage of the cost by Medicare Part D and most insurance organizations inhibits most facilities from using IDPN (1, 4, 19, 28, 30–32). In addition, the routine practice of using IDPN as a treatment for malnutrition has been limited by the availability of only a few randomized control studies which outline indications for use of IDPN to treat malnutrition and subsequent benefits from its use (4).

**Table 1:** Factors related to poor growth in CKD.

Poor nutrition intake
Age at onset of disease
Primary diagnosis
Race
Acidosis
Anemia
Decline in GFR/residual renal function
Growth factor abnormalities
Bone disease

### Definition of intradialytic parenteral nutrition

IDPN is a non-invasive method of providing nutrition to malnourished hemodialysis patients that has been in practice since the 1980s. It is infused through the hemodialysis access during the HD treatment via the venous limb to decrease dialyzer clearance of the amino acid. The volume is removed concurrently via ultrafiltration over the course of the HD treatment. Previous studies have shown IDPN to be an effective treatment for malnutrition in children and adults on hemodialysis (6, 7, 28). The 2009 KDOQI Guidelines recognize IDPN as an effective treatment for pediatric hemodialysis patients (16).

## Initiating IDPN

Before initiating IDPN, it is important to assess the patient's current nutrition status, dietary intake, and the reason for deficiencies (3–7, 9, 11, 16).

1. Oral Diet: Is the patient consuming adequate nutrition to meet 100% nutrition needs?
2. Oral Supplement: Is the patient prescribed an oral supplement? Is the patient adherent to intake of the supplement or does the patient refuse intake?
3. Enteral Supplement: Is the family adherent to providing the supplement? Does the patient tolerate an adequate amount of formula to meet nutrition needs?
4. Parenteral Nutrition: Is the patient already on partial/full TPN? TPN may be used in patients with gastrointestinal disease; however it may be challenging to meet 100% nutrition needs due to fluid restriction or poor ultrafiltration tolerance.

Various protocols for initiating IDPN have been reported (1–3, 5, 9, 16, 28). At Texas Children's Hospital Renal Dialysis Unit, IDPN is indicated if 2 or more of the below criteria are met:

- >10% loss of body weight in 3 months in a patient <90% IBW
- Unable to meet nutritional needs with enteral feeds
  - Fluid restriction
  - Trials to increase enteral feeds fail
  - Gastrointestinal disease (required for Medicare reimbursement for IDPN without objective data of malnutrition)
- Clinical signs of malnutrition
  - Serum albumin <3.5 mg/dL
  - Low nPCR <1 g/kg/day

Often times, however, the clinical judgment of the physician and/or dietitians may be used to determine risk and need for IDPN. Weight loss and poor tolerance of enteral feeds are clear indications. If weight loss and feeding tolerance criteria are not met, our studies have shown nPCR to be a better predictor of malnutrition risk and imminent weight loss when compared to serum albumin (6–8).

Components and Dosing (see IDPN prescription below).

There are 3 main components to IDPN: carbohydrate (CHO), protein, and fat. The recommended CHO component is 70% dextrose solution. The purpose of the CHO is to allow for protein-sparing of the amino acids and help prevent catabolism. It is dosed at 5–9 mg CHO/kg/min of HD time. The recommended protein is 15% amino acid to minimize the volume needed to provide adequate protein supplementation. Protein is dosed at 1.3 g/kg/treatment. The recommended lipid component is 20% egg-based solution used to minimize the volume infused and maximize caloric intake. Lipid is dosed at 25% of the IDPN (CHO and protein) volume. These dosing parameters are similar to other published prescriptions (1, 3, 4, 9, 11, 28, 31).

## Goals of IDPN therapy

The primary goal of IDPN therapy is to improve nutrition status by aiding true weight gain and hence improvement in BMI (1, 2, 16). Weight gain may occur by an increase in oral intake or supplementation to meet full nutrition needs. As a result of weight gain, the secondary goals of improving nPCR and serum albumin are subsequently also achieved. The expected time frame to reach these goals is 6 weeks to 6 months post-initiation of therapy. IDPN is only a supplement. It provides a significant amount of protein, but only a small percentage of the caloric needs. Therefore, the time to respond to therapy will vary based on access to food and home diet regimen. Adequate caloric intake is crucial to allow appropriate/catch-up weight gain. Once the goals are met, IDPN may be discontinued with a goal of weight/growth maintenance with oral/enteral supplementation alone (1, 5–7).

Krause et al. evaluated the effect of IDPN in four pediatric HD patients (9). The results were promising although not significant. The children gained weight and had an improvement in BMI, %IBW (73–88% to 79–90%), and oral intake [5–63 to 35–177 kcal/kg/day) by the time IDPN therapy was completed 3 months later. In studies at Texas Children's Hospital, IDPN was found to be an effective treatment for malnutrition in HD (Table 2). In the first study, 3 adolescent patients at final height were provided IDPN (6). Within 6 weeks of therapy, they all had a significant improvement in nPCR and all 3 gained weight: 2 reached IBW and 1 reached 90% IBW. In the second study, 8 patients who had lost >10% usual body weight and were <90% IBW were provided IDPN (7). In the initial 5 months of therapy, 5 of the 8 gained weight, 1 stopped losing weight, and 2 continued to lose weight. Although there were improvements in weight, BMI and nPCR, they were not significant. An assessment into the etiology for malnutrition revealed 5 patients had organic causes and 3 psychosocial causes. When analyzed by etiology, there was a significant improvement in nPCR and positive, although not statistically significant, improvement in weight, and BMI with IDPN therapy in the 5 with organic causes for malnutrition. These 5 patients eventually reached IBW with IDPN therapy. In conclusion, IDPN proved to be an effective treatment for organic causes of malnutrition. The study however, also highlighted that it may not be an effective treatment for those with psychosocial issues and emphasized the need to consider psychological intervention as well when managing malnutrition.

**Table 2:** IDPN effects on nPCR, sAlbumin, and Anthropometrics.

	**TCH Study #1 (****6****)**			**TCH Study #2 (Organic) (****7****)**		
	**Pre-IDPN**	IDPN*	***P*-value**	**Pre-IDPN**	IDPN*	***P*-value**
Mean nPCR (g/kg/d)	1.05 ± 0.36	1.35 ± 0.37	<0.05	1.07 ± 0.34	1.38 ± 0.47	0.03
Mean sAlbumin (g/dL)	3.7 ± 0.8	3.8 ± 0.6	NS	3.6 ± 0.61	3.5 ± 0.52	NS
Mean % ΔWt	−0.6 ± 2.7	1.8 ± 2.1	<0.02	−1.10 ± 3.3	0.10 ± 5.7	NS
Mean % ΔBMI	−1.3 ± 2.7	1.3 ± 2.1	<0.02	−1.06 ± 3.3	0.04 ± 5.7	NS
Mean spKt/V	1.49 ± 0.29	1.43 ± 0.18	NS	1.36 ± 0.28	1.26 ± 0.19	NS

* *All patients provided 1.3 g protein/kg/treatment, CHO, and fat per protocol (6, 7)*.

In a more recent study, Haskin et al. exclusively provided lipids (IL) based on the premise that IL therapy would spare protein degradation and promote positive nitrogen balance (33). They found significant improvements in serum albumin (3.2 ± 0.5 to 3.6 ± 0.4, *p* = 0.02), pre BUN (53 ± 11 to 66 ± 19, *p* = 0.03), and nPCR (0.98 ± 0.2 to 1.2 ± 0.3, *p* = 0.03), all indicating improved nutritional status. The increase in the pre BUN indicates increased appetite and intake likely associated with overall improved well-being. In addition, the cohort demonstrated a 5% weight gain (37 kg ± 16.3 to 38.7 kg ± 16.2, *p* = 0.04) during IL therapy without changes in triglyceride or cholesterol levels. They concluded that exclusive IL therapy was an effective and more economical treatment ($30/IL treatment vs. $170/total IDPN treatment) for improving nutritional status.

A summary of advantages and disadvantages of IDPN are presented in Table 3 (4, 5, 11, 29).

**Table 3:** Advantages and disadvantages of IDPN therapy.

**Advantages**	**Disadvantages**
Does not require separate central venous catheter Provides a significant amount of protein in a short period If it prevents hospitalization, costs are lowered and QOL is improved. Extra fluid and minerals are ultrafiltered during HD Convenient source of nutrition for the patient as requires little effort from the patient Less costly than TPN or hospitalization for malnutrition complications	Not a sole source of nutrition May not reverse malnutrition in non-organic cases Cost Extra fluid difficult to ultrafilter in an otherwise already fluid overloaded patient

## Parameters to monitor

Once on IDPN, tolerance must be monitored just as one would for any other type of feeding therapy, whether enteral or parenteral [Table 4; (1, 6, 7, 11, 16)]. With nutritional supplementation with IDPN/IL, patients are at risk for development of hyperglycemia, hypokalemia, hypophosphatemia, and hyperlipidemia.

**Table 4:** Elements to monitor on IDPN.

Glucose	Monitor pre-IDPN infusion, 1-h into the IDPN infusion, and post-IDPN infusion during the first week of starting IDPN or any changes made in dextrose rate, then monthly. If serum glucose >300 mg/dL, give insulinto keep serum glucose <200 mg/dL.
Triglycerides	Monitor pre-lipid infusion for the first 2 infusions, then monthly. If 50% rise above baseline level, discontinue lipids.
Potassium, phosphorus	Monitor for low levels due to increased insulin levels in the blood.

### Hyperglycemia

Hyperglycemia can occur as a consequence of excessive glucose infusion, a reaction to physiological stress, or as an adverse reaction to steroid therapy (29). The glucose infusion rate (GIR) at the maximum dose of carbohydrate prescription in IDPN is 9 mg/kg/min which is within the American Society for Parenteral and Enteral Nutrition (A.S.P.E.N.) guidelines (34). Krause et al. experienced issues with hyperglycemia and hypophosphatemia (9) which was attributed, respectively, to the high GIR and the consequential increase in insulin release following the hyperglycemia.

### Hypokalemia and hypophosphatemia

Malnutrition can predispose a patient to hypokalemia and hypophosphatemia if refeeding syndrome occurs (34). Refeeding may occur with a high dose of dextrose infusion. The IDPN protocol presented in this paper however provides a low dose of carbohydrate.

### Hyperlipidemia

A.S.P.E.N. and German guidelines recommend a maximum dose of fat at 1–2 g/kg/day for lipids (3, 34). The protocol presented in this paper doses IL at ~0.5 g fat/kg/day. IL are provided only during HD which for most patients is every 2 days allowing time for the lipids to clear (or to be metabolized). A.S.P.E.N. recommends holding or reducing IL for triglycerides levels >250 mg/dL. The guidelines presented in this paper define intolerance as a 50% increase from baseline since triglyceride levels may be slightly elevated in patients with ESRD (16).

In addition, it is important to assess for drug-nutrient interactions. If there is a potential interaction, it is imperative to work with the physician and/or pharmacist to ensure medications and nutrition are provided safely and adequately (29, 34). All monitoring recommendations presented are within A.S.P.E.N. recommendations. Routine monthly lab monitoring for dialysis patients meets both KDOQI and A.S.P.E.N. guidelines (16, 34).

## Conclusion

IDPN is an effective therapy for treatment of malnutrition in pediatric HD patients. Exclusive lipid therapy has promise. More studies however are needed to assess the effects of IDPN on height/growth failure, other known metabolites associated with increase malnutrition risk, and protein utilization. In addition, studies assessing the potential use of carnitine and other vitamins and trace mineral supplements in IDPN to enhance nutrient provision should be considered to assess other potential benefits of IDPN.

## IDPN prescription

Carbohydrate (CHO) Component Calculation: Prescribe 3–9 mg/kg of Dextrose 70% solution

(_^*^_) mg × patient weight (___) kg × dialysis run time (___) minutes = (___) g CHO
  1000
  ^*^Note: the range is 3-9 mg
(___) g carbohydrates__ = (___) mL of Dextrose 70% (**A**)
  0.7 g carbohydrates/mL

Protein Component Calculation: Prescribe 1.3 g/kg/day of Amino Acid 15%

1.3 g protein/kg/day × patient weight (___) kg = (___) g of protein
(___) g of protein = (___) mL of Amino Acid 15% (**B**)
  0.15 g of protein/mL

Total IDPN Volume and Infusion Rate: Add total ml of Dextrose 70% + Amino Acid 15% and divide by total hours of prescribed HD time

1. Total IDPN Volume: Dextrose 70% (A) + Amino Acids 15% (B) = (___) mL
2. Infusion Rate IDPN: (___) mL Dextrose 70% (A) ± (___) mL Amino Acids 15% (B) = (___) mL/h IDPN
  (___) hours dialysis time

Intralipid Component Calculation: The intralipid calculation is based on the total IDPN volume. Intralipids should start with 0.5 ml/min (30 ml/h) for the first 30 min of initial infusion of intralipids. The maximum rate of intralipid infusion should not exceed 62.5 ml/h. This calculation method provides ~0.5 g fat/kg/treatment.

Intalipid (IL) Component Calculation:
  [(___) mL Dextrose 70% (A) + (___) mL Amino Acids 15% (B)] × 0.25 = (___) mL of IL20%
  Intralipid Infusion Rate:
(___) mL of Intralipid 20% = (___) mL/h Intralipid 20%
  (___) hours of dialysis time

## Author contributions

The author confirms being the sole contributor of this work and has approved it for publication.

### Conflict of interest statement

The author declares that the research was conducted in the absence of any commercial or financial relationships that could be construed as a potential conflict of interest. The handling editor declared a shared committee, though no other collaboration, with one of the authors MJ at time of review.
